# Mitotic control of human papillomavirus genome-containing cells is regulated by the function of the PDZ-binding motif of the E6 oncoprotein

**DOI:** 10.18632/oncotarget.14469

**Published:** 2017-01-03

**Authors:** Elizabeth K. Marsh, Craig P. Delury, Nicholas J. Davies, Christopher J. Weston, Mohammed A.L. Miah, Lawrence Banks, Joanna L. Parish, Martin R Higgs, Sally Roberts

**Affiliations:** ^1^ Institute of Cancer and Genomic Sciences, University of Birmingham, Birmingham, United Kingdom; ^2^ Institute of Immunology and Immunotherapy, University of Birmingham, Birmingham, United Kingdom; ^3^ International Centre for Genetic Engineering and Biotechnology, Padriciano, Trieste, Italy; ^4^ Faculty of Medicine, Dentistry and Health, University of Sheffield, Sheffield, United Kingdom

**Keywords:** human papillomavirus, E6, PDZ proteins, HPV life cycle, mitosis

## Abstract

The function of a conserved PDS95/DLG1/ZO1 (PDZ) binding motif (E6 PBM) at the C-termini of E6 oncoproteins of high-risk human papillomavirus (HPV) types contributes to the development of HPV-associated malignancies. Here, using a primary human keratinocyte-based model of the high-risk HPV18 life cycle, we identify a novel link between the E6 PBM and mitotic stability. In cultures containing a mutant genome in which the E6 PBM was deleted there was an increase in the frequency of abnormal mitoses, including multinucleation, compared to cells harboring the wild type HPV18 genome. The loss of the E6 PBM was associated with a significant increase in the frequency of mitotic spindle defects associated with anaphase and telophase. Furthermore, cells carrying this mutant genome had increased chromosome segregation defects and they also exhibited greater levels of genomic instability, as shown by an elevated level of centromere-positive micronuclei. In wild type HPV18 genome-containing organotypic cultures, the majority of mitotic cells reside in the suprabasal layers, in keeping with the hyperplastic morphology of the structures. However, in mutant genome-containing structures a greater proportion of mitotic cells were retained in the basal layer, which were often of undefined polarity, thus correlating with their reduced thickness. We conclude that the ability of E6 to target cellular PDZ proteins plays a critical role in maintaining mitotic stability of HPV infected cells, ensuring stable episome persistence and vegetative amplification.

## INTRODUCTION

Of the more than 200 human papillomavirus (HPV) types that are known to infect humans, twelve have been defined as human carcinogens (HPV16, 18, 31, 33, 35, 39, 45, 51, 52, 56, 58 and 59), with another twelve types that are closely related to the carcinogenic viruses, defined as “probably” or “possibly carcinogenic” [1]. These so-called high-risk (HR) viruses are closely associated with the development of epithelial cancers of the anogenital and oropharyngeal tracts [2–4]. The continuous expression of the HPV early proteins E6 and E7 is one of the defining features of HPV driven carcinogenesis and thus, they are attractive targets for therapeutic intervention of these tumours. The ability of E6 and E7 proteins to deregulate host mechanisms of cell cycle control, senescence and survival can lead to cellular immortalization, and the development of genetically unstable cells that are at increased risk of oncogenic transformation [5]. The key biological activities of the E6 oncoprotein that contribute to carcinogenesis include the capacity to inactivate the p53 tumor suppressor protein, activation of telomerase and the targeting of specific cellular proteins containing PDZ (PSD95/DLG1/ZO-1) domains (referred to as PDZ proteins) [6].

Binding between PDZ proteins and E6 is mediated by a C-terminal class I PDZ domain-binding motif (PBM) that is only found in E6 proteins of the HR-HPV types, suggesting that the motif acts as an oncogenic signature. Indeed, the E6 PBM was shown to be necessary for the morphological transformation and the induction of tumorigenesis of rodent cell lines [7]. Moreover, in a transgenic mouse model of cervical carcinogenesis, the presence of the HPV16 E6 PBM function enhanced tumor development, affecting both tumor size and area of tumor invasion [8]. In mammalian keratinocytes, the E6 motif has been linked to cell proliferation [9, 10] and whilst not necessary for the immortalization of these cells, it has been shown to contribute to epithelial-mesenchymal transition and anchorage–independent cell growth; two acquired phenotypes that are linked to invasive and metastatic growth of tumors [11–14].

The interaction between E6 and PDZ proteins most often leads to ubiquitin-mediated degradation of the host protein via the proteasome [15], although this is not the case for all PDZ targets [16, 17]. Phosphorylation of the E6 PBM has been shown to negatively regulate the interaction with PDZ protein partners and confers the ability of the E6 protein to interact with a number of 14-3-3 isoforms [18, 19]. Many of the PDZ proteins that associate with E6 have roles in governing cell polarity, including apico-basal epithelial polarity and asymmetric cell division. In fact, core components of all the apico-basal polarity modules (Crumbs, Par and Scribble) are targets of the E6 domain. These include PALS1-associated tight junction protein (PATJ) of the Crumbs complex [20], PDZ portioning defective 3 protein (PAR3) of the Par complex [16] and the human homologues of the *Drosophila* tumor suppressor proteins, discs large 1 (DLG1) and scribble (hSCRIB), both of the Scribble complex [7, 15, 21, 22]. Other PDZ targets of E6 include members of the MAGI family of proteins, which are also linked to polarity [23, 24], and the non-receptor tyrosine phosphatases PTPN3 and PTPN13, that both have roles in regulating signal transduction pathways involved in cell proliferation, apoptosis, migration and intracellular trafficking [25, 26].

The function of the E6 PBM has been shown to play an essential role in the replication cycle of HR-HPV types. Our studies using organotypic raft cultures of primary human foreskin keratinocytes (HFK) transfected with a mutant HPV18 genome which expresses an E6 protein lacking the PBM (E6ΔPDZ), showed a deleterious effect of loss of this E6 function upon the productive phase (viral episome amplification and viral late gene expression) of the virus life cycle [27]. Further analysis of these cultures demonstrated that the defect in the productive cycle was linked to a reduction in cell proliferation in the upper layers of the stratified cultures. Both viral episome establishment and stable maintenance of the viral episomes were also compromised upon monolayer cell growth of the basal-like undifferentiated cells [27], observations that were supported by investigations of other HR-HPV types [28, 29]. Collectively, these studies indicate that the E6 PBM is critical for multiple stages of the infectious cycle of cancer–causing HPV types.

In this study, we have investigated in greater detail the impact of loss of the E6 PBM function on the growth of viral genome-containing keratinocytes. We show that this function of E6 is strongly associated with the maintenance of mitotic stability of episome-containing cells. In the absence of the E6 PBM, cells develop severe mitotic abnormalities and accrue genomic instability. Thus, this function of E6 is vital for episome maintenance and vegetative replication, potentially by acting to safeguard mitotic integrity of HPV-infected cells.

## RESULTS

**Figure 1:**
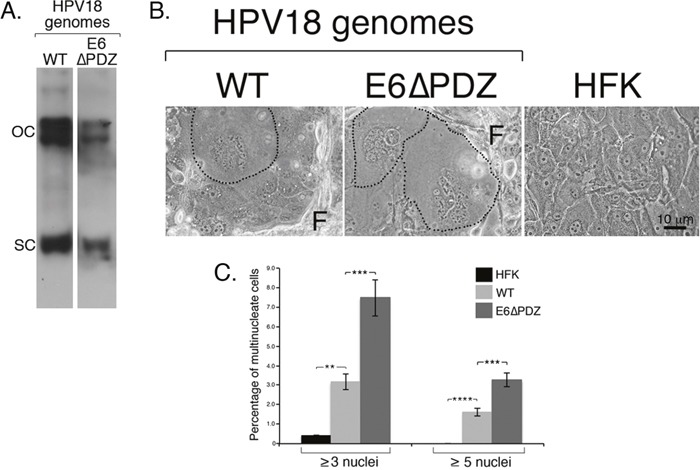
Loss of E6 PBM function is associated with enhanced frequency of nuclear atypia of viral genome-containing cells **A**. Southern analysis confirmed presence of HPV18 wild type (WT) and mutant (E6ΔPDZ) episomes migrating as supercoils (SC) and open circles (OC) in cells; the mutant genomes establish at a lower copy number than the WT genomes as shown previously (the images of the two lanes were taken from the same Southern blot at a single exposure) [27]. **B**. Phase contrast images of cells grown in monolayer culture, including untransfected donor cells (HFK), with fibroblast feeder cells (F). HPV18 genome-containing cells showing nuclear atypia (multinucleation) are identified (black dotted line). **C**. Bar graph, percentage of cells containing multiple nuclei; data (mean ± standard error of the mean) derived from random fields of view and cells from three independent donors. Student *t* test was used to determine the significance (**, P<0.01; ***, P<0.001; ****, P<0.0001). Total number of cells counted: HFK, 2000 cells; WT, 1400 cells; E6ΔPDZ, 1060 cells.

As described previously, we genetically engineered the wild type (WT) HPV18 genome to replace codons at positions 155 and 156 of the E6 protein with translation termination codons, creating the mutant genome E6ΔPDZ [27]. Thus, the E6 protein expressed from the mutant genome lacked the C-terminal four amino acids constituting the PBM (^155^ETQV^158^). The deletion of the HPV18 E6 PBM negates the targeting of PDZ proteins but does not interfere with other E6 functions, including the ability of the E6 protein to degrade the p53 tumor suppressor protein [27]. We established HPV18 genome-containing cell lines by transfecting primary human foreskin keratinocytes (HFK) from three different donors (#1, #2 and #3) with the WT genome or the mutant genome. Both the WT and E6ΔPDZ genomes replicated as extrachromosomal plasmids in these cells (Figure 1A); although E6ΔPDZ was present at reduced copy number in comparison to the WT episomes, in agreement with our previous findings [27]. Notably, the E6ΔPDZ mutation does not adversely affect the levels of the E6, or the E7 proteins expressed in the HPV18 genome-containing cells grown in undifferentiated monolayer culture or in organotypic raft culture [27].

Since extended cell culture of E6ΔPDZ cell lines often leads to a sharp reduction in maintenance of episomes and viral DNA integration [27], only cell passages (≤ 35 cell population doublings) prior to these viral genetic events (as determined by Southern analysis) were used in this study.

### Deletion of the E6 PBM is associated with enhanced frequency of nuclear atypia of HPV18 genome-containing cells

By using bright-field microscopy, it was noticeable that in monolayer cell growth conditions, in comparison to the untransfected donor HFK, both the WT and E6ΔPDZ genome-containing cell cultures were characterized by the presence of cells with multiple, and often misshapen nuclei (Figure 1B). Quantification of HFK cells showed that 0.43% of cells had this aberrant multinucleated morphology (≥ 3 nuclei/cell), whereas the percentage of multinucleated cells increased by 7-fold in WT genome containing cells (3.15%) (Figure 1C). Interestingly, multinucleation of cells harboring the E6ΔPDZ genomes was enhanced further (17-fold change; 7.47% of cells) (Figure 1C). Furthermore, cells containing the E6ΔPDZ genomes showed a 2-fold increase in frequency of cells with high levels of multinucleation (≥ 5 nuclei/cell) in comparison to those carrying the WT genomes, increasing from 1.61% (WT) to 3.26% (E6ΔPDZ). These data demonstrate that the replication of HR-HPV episomes in primary keratinocytes leads to perturbation of cell division, in agreement with previous suggestions [30–32]. Moreover, our analysis also suggests that loss of the E6 PDZ binding function further increases the deleterious effect of the virus on coordinated cell division.

### Live cell microscopy reveals an increase in frequency of cells undergoing abnormal mitosis in E6ΔPDZ cell cultures

**Figure 2:**
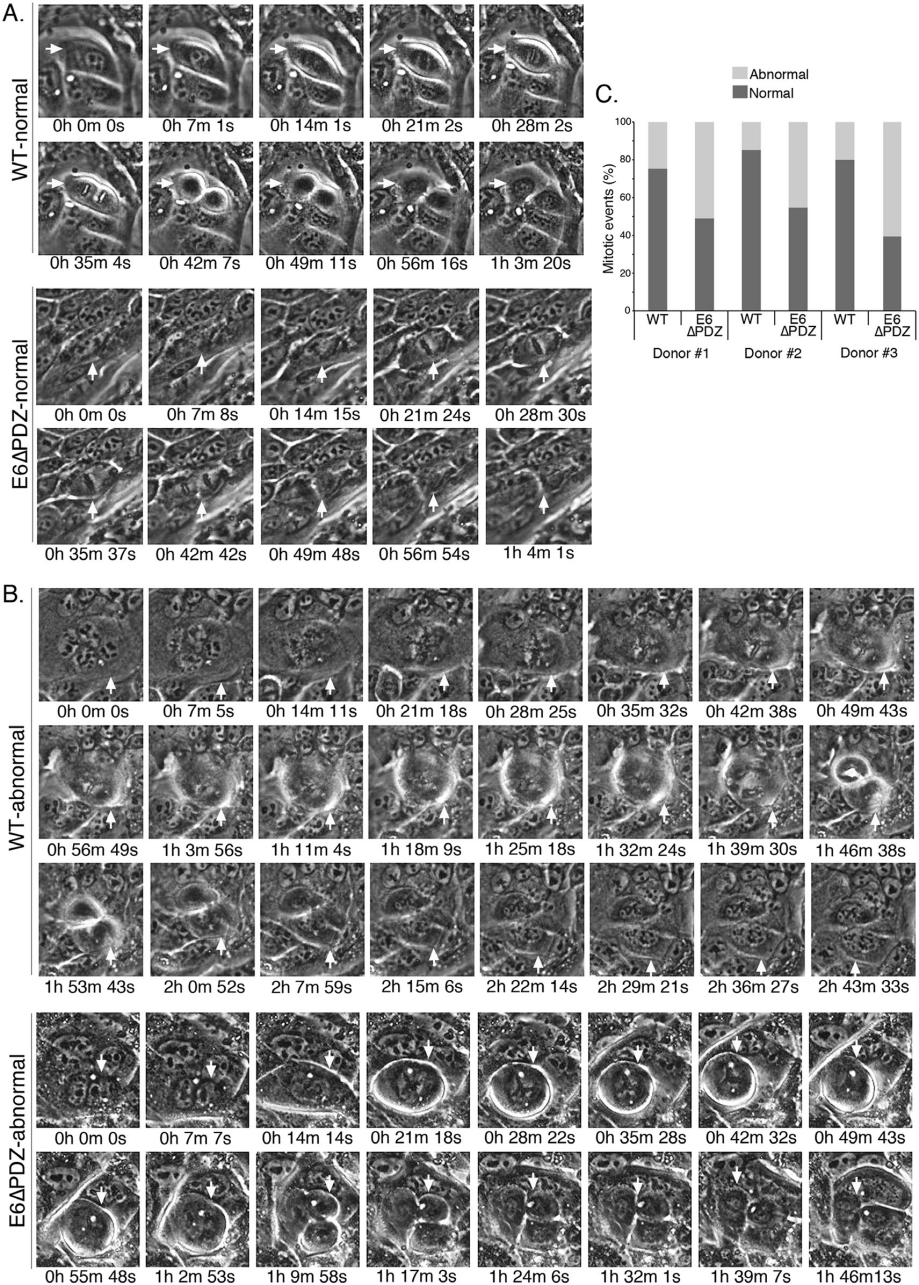
Live cell microscopy shows that loss of E6 PBM function is associated with an increase in abnormal mitoses in viral genome-containing cells Examples of normal **A**. and abnormal **B**. mitoses from the live cell microscopy in cell lines harboring the WT or the E6ΔPDZ genomes (movies given in Supplementary Figure 1). Cells undergoing mitosis are identified with a white arrow and the time to complete mitosis is given. Note that in these examples of abnormal mitosis, the event is multipolar and the time taken to completion is much longer than for completion of normal cell division. **C**. Bar graph, percentage of mitoses that were normal or abnormal in each of the three donor backgrounds. Mitoses defined as normal if bipolar, had visible and equal separation of condensed chromatin and the time to completion of mitosis was not excessive; otherwise the mitotic event was scored as abnormal. Number of colonies counted was in the range of 14 to 23 and for each of the cell lines between 90-308 mitoses counted.

To investigate whether the increase in multinucleated cells observed upon loss of the E6 PBM was due to increased mitotic errors, we next examined mitotic progression of the cells by live cell imaging of asynchronous monolayer cell cultures. Cells carrying either WT or E6ΔPDZ genomes were plated at low cell density and once individual cell colonies were visible they were imaged for 16 h by phase contrast microscopy, using the Cell-IQ system. The resulting time-lapse movies were subsequently analyzed to determine the number of mitotic events within individual colonies, and each mitotic event classified as ‘normal’ or ‘abnormal’ according to defined criteria (e.g. time taken from mitotic entry to daughter cell separation, mitotic spindle polarity, visible and equal separation of condensed chromosomes). Images of representative examples of normal and abnormal mitotic cell divisions are given in Figure 2A and 2B respectively, and movies are shown in Supplementary Figure 1. In order to eliminate any donor keratinocyte effects, the analysis was performed on cell lines established in the three different keratinocyte backgrounds.

The distributions of normal and abnormal mitoses in the HPV18 genome–containing cells are shown in Figure 2C. The majority of mitotic cells carrying the WT genomes underwent normal cell division, with less than 25% (a range of 14.5% to 24.6% between the three donors) undergoing an aberrant division. However, in cells carrying the E6ΔPDZ genomes, the proportion of mitotic cells undergoing abnormal cell division increased markedly across all three donors (a range of 45.5% to 60.8%). Moreover, we also noted that in E6ΔPDZ-containing cell lines, even cells with elevated numbers of nuclei (>15) entered mitotic division (Supplementary Figure 2). In the examples given in Figure 2A, the time to complete a normal bipolar mitotic division for both WT and E6ΔPDZ genome-containing cells is just over 1 h, with no noticeable differences in the time taken to complete mitosis between cell lines. However, the abnormal WT and E6ΔPDZ divisions are multi-radial and time to completion is extended to over 1 h 45 min (Figure 2B). Taken together, these data indicate that in the context of whole HR-HPV genomes, loss of E6 PBM function is associated with an increase in the frequency of abnormal mitotic events.

### Loss of E6 PBM function is associated with an increase in spindle-associated abnormalities in late stage mitosis

Next, we wanted to further characterize the mitotic defect observed in the E6ΔPDZ genome-containing cells. Therefore, cells were grown in monolayer cell culture on glass coverslips, fixed and stained for the mitotic spindle protein alpha-tubulin and counterstained with the DNA dye DAPI. As before, mitotic cells were scored from random fields of view as to whether ‘normal’ or ‘abnormal’; the specific events were chromosome alignment on spindle and spindle polarity in metaphase; successful separation of chromosomes, spindle polarity in anaphase and telophase, the presence of anaphase or telophase bridges, and telophase spindle polarity.

**Figure 3:**
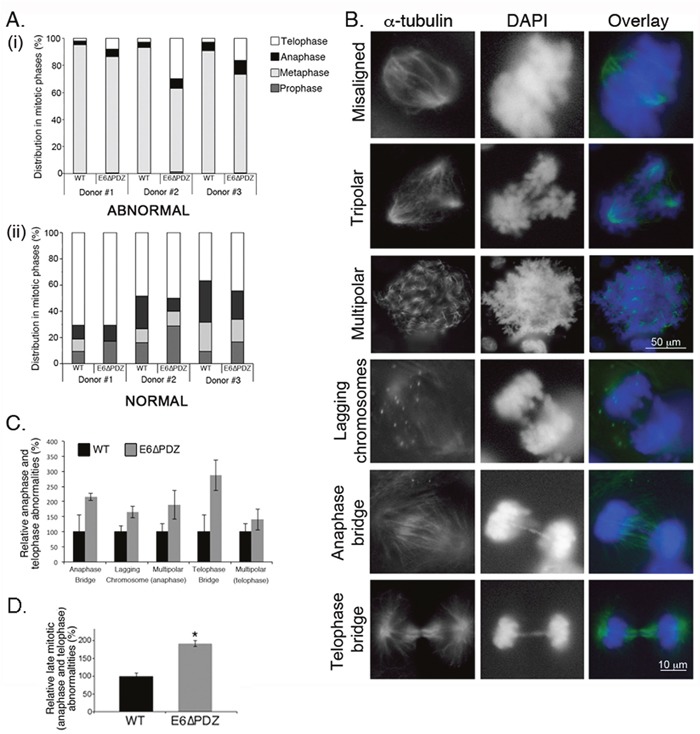
Loss of E6 PBM function leads to an enhancement in the frequency of abnormal cells progressing into anaphase and telophase **A**. Abnormal (bar graph i) or normal (bar graph ii) mitoses were scored in each stage of mitosis (prophase, metaphase, anaphase and telophase) by α-tubulin staining and counterstaining with DAPI. The data are derived from three independent experiments for each of the three donors and presented as a proportion of total abnormal (top graph) or normal (bottom graph) mitoses. For each experiment, a minimum of 200 cells per donor was counted. **B**. Examples of abnormal mitoses in HPV18 genome-containing cells showing spindle morphology (α-tubulin, green) and chromosomes (DAPI, blue). **C**. Individual mitotic abnormalities were scored by α-tubulin staining and counterstaining with DAPI. Only the data for anaphase and telophase abnormalities are shown. The data are derived from three independent experiments for each of the three donors, with a minimum of 100 abnormal mitotic cells scored for each experiment, and the data is shown as the mean (± standard error of the mean). The differences in the frequency of individual abnormalities between WT and E6ΔPDZ cells were not statistically significant. **D**. The frequency of the combined late (anaphase and telophase) mitotic abnormalities in the E6ΔPDZ genome containing cells shown relative to cells harboring the WT genomes. Student's *t* test was used to determine the significance (*, P<0.05).

Both WT and E6ΔPDZ cells were identified in prophase, metaphase, anaphase and telophase in all three individual donor backgrounds. In agreement with the findings from the live microscopy analysis (Figure 2), increased abnormal mitoses were observed in cells harboring the mutant E6ΔPDZ genomes (data not shown). Unlike the normal mitotic cells, which were mainly in anaphase-telophase, the majority of abnormal mitotic WT and E6ΔPDZ genome-containing cells were in metaphase (Figure 3A). Moreover, whilst very few of the cells carrying WT genomes had defects in anaphase and telophase, in comparison, the proportion of E6ΔPDZ cells with spindle abnormalities in both anaphase and telophase increased in all three donors (Figure 3A, i, and 3B). Thus, in donor #1, abnormalities in anaphase and telophase of E6ΔPDZ genome-containing cells increase by 2.7-fold, in donor #2 by 5.6-fold and in donor #3 by 3.0-fold, compared to cells harboring the WT genomes. These data suggest that whilst only a small proportion of cells harboring WT HPV18 genomes with metaphase abnormalities are able to progress through the mitotic cycle, a greater proportion of abnormal cells containing the mutant genome are able to move into anaphase and telophase. Examples of mitotic abnormalities detected in the HPV18 genome-containing cells are shown in Figure 3B.

To further define the underlying defects occurring in anaphase and telophase in the E6ΔPDZ cells, we next analyzed the frequency of specific mitotic abnormalities in anaphase (anaphase bridges, lagging chromosomes, spindle polarity) and telophase (spindle polarity, telophase bridge) (see Figure 3B for examples of these defects in E6ΔPDZ cells). From this analysis, it was shown that there was increased frequency of defects associated with anaphase and telophase in E6ΔPDZ genome-containing cells compared to the WT genome controls (Figure 3C). Indeed, the combined frequency of these abnormalities was statistically significant (p<0.05) relative to the cells harboring the WT genomes, although the difference in the frequency of any one abnormality did not reach statistical significance (Figure 3C and 3D). Together, these data suggest that loss of the E6 PBM function is linked to a spindle abnormality in the viral episome-containing cells and not a signaling defect associated with one specific mitotic abnormality. In conclusion, in HPV18 genome-containing cells, the loss of E6 PDZ binding resulted in a quantitative increase in mitotic defects persisting late into mitosis.

### Increased frequency of centromere-positive micronuclei in E6ΔPDZ genome-containing cells

**Figure 4:**
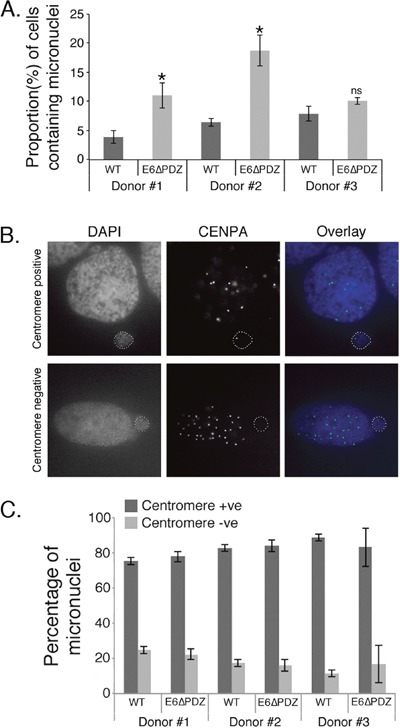
Increased micronuclei formation associated with E6ΔPDZ genome-containing cells **A**. The proportion of cells containing micronuclei was scored from DAPI-stained cells. The data were collected from three independent experiments, with a minimum of 200 cells for each donor scored on each occasion. The data are shown as the mean (± standard error of the mean), and in all three donors the frequency was increased in E6ΔPDZ genome-containing cells (*, p<0.05); ns, not significant). **B**. Images of examples of CENPA positive and negative nuclei; in overlay CENPA (green), nuclei (blue). **C**. The proportion of cells with micronuclei containing centromeres was determined by immunofluorescence staining for CENPA. The data were collected from three independent experiments, with a minimum of 200 cells with micronuclei scored for each experiment, and shown as the mean (± standard error of the mean).

Our findings that loss of the E6 PBM function led to an increase in the frequency of spindle abnormalities in anaphase and telophase suggested that cells harboring the E6ΔPDZ genomes would exhibit increased chromosome segregation defects. One consequence of these is the partition of chromosomes into micronuclei, whereby nuclear membranes form around lagging chromosomes and are visible in interphase cells as discrete extra-nuclear formations of enveloped DNA. Therefore, to investigate this possibility, the frequency of cells with micronuclei was determined in cells harboring the HPV18 genomes (Figure 4A). Micronuclei were observed in cells replicating either WT or E6ΔPDZ genomes, but the frequency of cells with micronuclei increased in the presence of E6ΔPDZ genomes (1.3 – 2.9 fold increase compared to WT genome-containing cells), although these increases only reached significance in two out of the three donors analyzed. These data therefore support our previous conclusion that the loss the E6 PBM function enhances the dysfunction of the mitotic apparatus.

Since micronuclei formation occurred in the presence of WT as well as the mutant genomes, it was important to establish if they arose through similar mechanisms. Micronuclei may arise from both chromosome segregation errors to give centromere-positive micronuclei; or through failed separation of chromosome fragments to produce centromere-negative micronuclei. Therefore, cells were stained with an antibody that detects the centromere protein, centromere protein A (CENPA) and micronuclei were then scored as CENPA-positive or –negative (Figure 4B). In both WT and E6ΔPDZ genome-containing cells the majority of micronuclei were CENPA–positive and this was consistent across the three different donor backgrounds, with no significant difference in the frequencies of the two populations between the different viral genomes (Figure 4C). These data indicate that the formation of micronuclei in WT or mutant genome-containing cells occurs through similar mechanisms, although this is enhanced in the absence of the E6 PBM function. Moreover, since the majority of micronuclei formed in HPV-containing cells did not contain acentric chromosomes, this suggests that these micronuclei arise from chromosome segregation errors, in support of our previous data (Figure 3).

### Increased aneuploidy of cells containing E6ΔPDZ genomes

Both numerical and structural chromosomal abnormalities are a frequent feature of HPV oncoprotein expressing cells; and the expression of both E6 and E7 proteins induce mitotic abnormalities in keratinocytes [31, 33]. If, as our data indicate, the loss of the PDZ binding function of E6 causes an increase in abnormal cells in anaphase and telophase, and elevated levels of micronuclei formation, then this would lead to an increase in the frequency of chromosomal abnormalities. To examine this hypothesis, interphase fluorescence *in situ* hybridization (FISH) using two chromosome-specific centromeric probes against chromosomes 10 and 12 was performed on cells harboring WT or mutant E6ΔPDZ genomes. For each cell line, three cell passages were investigated (approximately 16 - 24 population doublings between the first and third passages tested). Importantly, since extended cell culture of E6ΔPDZ cell lines often leads to loss of episomes and viral DNA integration, only cell passages prior to these viral genetic events (as determined by Southern analysis [27]) were subjected to FISH analysis.

**Figure 5:**
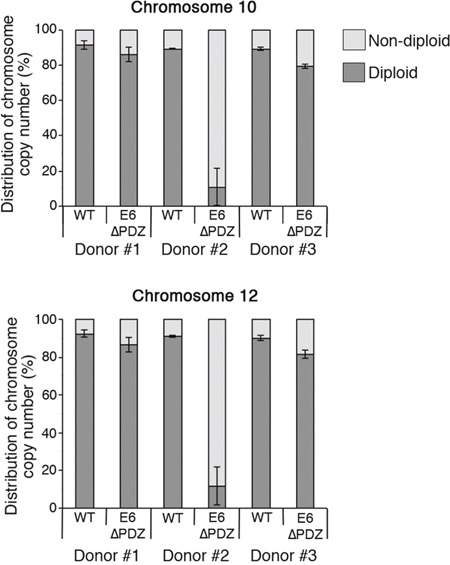
Increased aneuploidy of E6ΔPDZ genome-containing cells Interphase fluorescence *in situ* hybridization using centromere enumeration probes for chromosomes 10 and 12. The data were collected from three different cell passages, with a minimum of 200 cells with single nuclei scored for each cell passage and given as the percentage of nuclei with diploid or non-diploid chromosome copy number (mean ± standard error of the mean). The statistical significance of the increase in non-diploid copy number was: donor#1, ns; donor#2, p<0.01 for chromosome 10 and 12; donor#3, p<0.01 for chromosome 10 and p<0.05 for chromosome 12.

Figure 5 shows the distribution of diploid and non-diploid chromosome copy number in interphase cells from the three cell passages of both WT and E6ΔPDZ genome-containing cells. In comparison to WT genome carrying cells, there was an increase in the frequency of E6ΔPDZ cells with a non-diploid genome content and this was observed across the three donor backgrounds (Figure 5). The level of increase in aneuploidy is statistically significant in two of the donors (donor #2, p<0.01 for both chromosomes probes; donor #3, p<0.01 for chromosome 10 and p<0.05 for chromosome 12). Whilst the frequency of E6ΔPDZ cells with non-diploid chromosome content is increased in all donors, it is markedly enhanced in one of the donors (donor #2), by 8.4-fold (chromosome 10) and 10.0-fold (chromosome 12) compared to WT cells, suggesting donor-specific variation. There was no marked change in the frequency of cells with non-diploid chromosome copy number upon population-doubling of all the cell lines (*i.e*. comparison between the first and third cell passages, data not shown). Both loss and gain of chromosome copy number occurred in the presence of WT and E6ΔPDZ genomes. However, the prevailing trend across all three donors was increased frequency of cells with >4 chromosome copy number in cells carrying the mutant HPV18 genome defective in PDZ targeting (Table 1).

**Table 1:** Relative fold change in chromosome copy number in E6ΔPDZ genome-containing cells compared to cells harboring WT genomes

Donor	Chromosome	Copy number loss	Copy number gain	Copy number gain >4
**# 1**	1012	4.60.7	1.32.0	2.12.6
**# 2**	1012	0.91.1	9.811.4	24.369.4
**# 3**	1012	1.61.7	1.91.9	6.51.8

### Loss of E6-PDZ binding correlates with a change in the distribution of mitotic cells between the basal and suprabasal layers of organotypic raft cultures

We have previously shown that upon organotypic raft growth, the stratified cultures containing the E6ΔPDZ mutant genome showed decreased hyperplastic morphology relative to the WT genomes; they do not show the characteristic thickening associated with replication of WT genomes and there was a reduction in expansion of S phase-competent cells in the upper suprabasal layers [27]. Since our data thus far suggest that replication of the E6ΔPDZ mutant genomes is associated with mitotic abnormalities linked to spindle defects, we next wanted to determine if mitotic defects also occurred upon stratification of the E6ΔPDZ genome-containing cells, using α-tubulin staining to identify mitotic cells in sections of the organotypic rafts.

**Figure 6:**
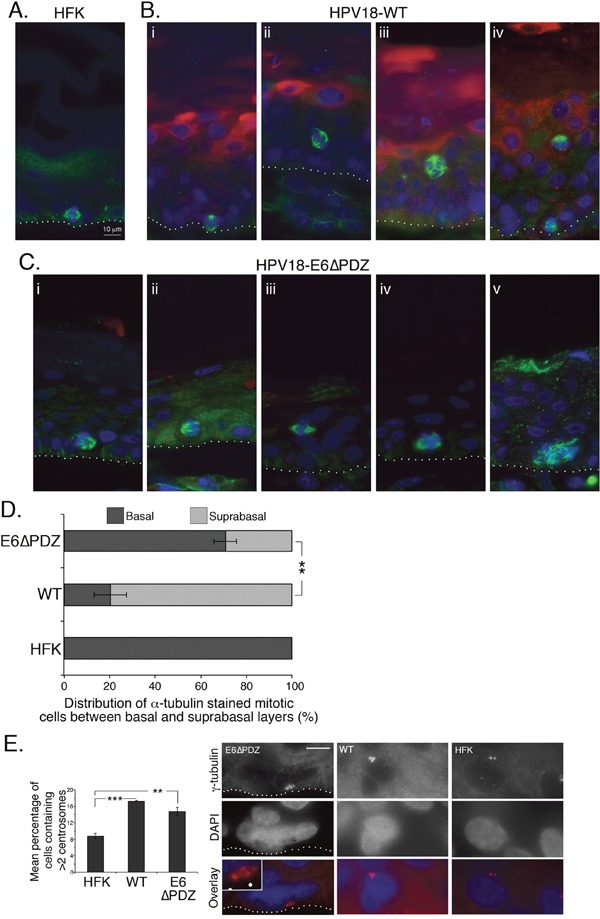
Loss of E6 PBM function is associated with an alteration in the distribution of α-tubulin stained mitotic cells between basal and surpabasal cell layers of organotypic rafts Sections of organotypic raft cultures of **A**. untransfected HFK, **B**. HPV18 WT genome-containing cells and **C**. cells containing E6ΔPDZ genomes, were stained for α-tubulin (green), HPV18 E4 (red), and DNA (blue). Examples given in B and C were taken from multiple donors. The dotted white line in A, B and C indicates the junction between the basal cell layer and fibroblast embedded collagen matrix. **D**. The distribution of mitotic cells between the basal and suprabasal cell layers of organotypic rafts shown as a percentage of total α-tubulin stained mitotic cells (± standard error of the mean). Data were collected from three donors, and for each donor five sections of organotypic raft were analyzed (full thickness and entire length). Numbers of α-tubulin stained mitotic cells counted were 47 (HFK), 525 (WT) and 230 (E6ΔPDZ). Student's t test was used to determine significance (**, p<0.01). **E**. Sections were stained with γ-tubulin to visualize centrosomes and cells with visible centrosomes were scored for centrosome number. Rafts prepared from three different donors were analyzed; numbers of cells scored were: 1772 (HFK), 4022 (WT) and 2758 (E6ΔPDZ). Student's t test was used to determine significance (**, p<0.01; ***, p<0.001). Shown are examples of cells in E6ΔPDZ, WT rafts with >2 centrosomes, and an HFK raft with 2 centrosomes (red, γ-tubulin; blue, DAPI). The E6PDZ centrosomes are shown enlarged in the inset and the dotted white line indicates the junction between the basal cell layer and fibroblast embedded collagen matrix. Scale, 10 μm (inset 18.5 μm).

In organotypic rafts of untransfected keratinocytes, few cells undergo mitosis and these are always restricted to the basal layers (Figure 6A). In contrast, mitotic cells showing α-tubulin staining of the spindle were present in both the basal and suprabasal layers of rafts generated from the WT (Figure 6B, panels i, ii and iv) and the E6ΔPDZ (Figure 6C, panels i – iii) genome-containing cells. However, the majority of mitotic WT cells were observed in the suprabasal compartment (basal, 20%; suprabasal 80%), consistent with the expansion of these epithelial layers (Figure 6D), whereas in rafts generated from cells harboring the E6ΔPDZ genomes, a significantly (p<0.01) greater proportion of mitotic cells remained in the basal layer compared to the WT raft cultures (basal, 71%; suprabasal 29%, Figure 6D). In the WT genome containing rafts, where polarity of the spindle was evident, it was most often bipolar (Figure 6B, panels i, ii and iv), although occasional cells with multipolar spindles were present (Figure 6B, panel iii). Interestingly, the polarity of the spindle could often not be determined in the E6ΔPDZ raft cultures, and where two poles were visible these were abnormal in appearance (Figure 6C, panel iii). Moreover, multipolar mitoses were present in E6ΔPDZ rafts (Figure 6C, panels iv and v).

HR-HPV driven host genomic instability has been linked also to the induction of centrosome abnormalities by the viral oncoproteins [34, 35]. Significantly, abnormal centrosome amplification is observed in stratified cultures of HPV16 episome-containing cells, indicating that this centrosome defect occurs during the normal replication cycle of the virus [30]. Thus, we sought to determine if centrosome amplification occurred in stratified cells harboring the HPV18 genomes by staining raft sections with γ-tubulin to visualize the centrosomes. The number of centrosomes in centrosome-positive cells was scored and cells with >2 centrosomes were quantified (Figure 6E). In the presence of WT genomes there was a significant increase in cells containing >2 centrosomes compared to the untransfected donor keratinocytes (17.24% WT vs 8.79% HFK). Notably, these data concur with the findings derived from HPV16 genome-containing rafts [30]. A similar proportion of cells (14.73%) with >2 centrosomes occurred in the stratified cultures of E6ΔPDZ genome-containing cells (Figure 6E). Thus, the exacerbation of mitotic dysregulation that we observe in HPV18 genome-containing cells upon loss of the E6 PDZ binding function is independent of the virus-induced effects upon the centrosomes.

## DISCUSSION

Our study has investigated the role of the E6 PBM in a physiologically significant HPV life cycle model based on primary human epidermal keratinocytes harboring episomal forms of the HPV18 genome [27, 36]. We have shown that a population of the keratinocytes harbouring the HR-HPV episomes undergo abnormal mitosis and that the size of this population increased upon loss of the four amino acids that constitute the HPV18 E6 PBM. In the presence of the WT genomes, the majority of cells that undergo abnormal mitosis exhibited metaphase associated spindle defects, as determined by α–tubulin-staining, and very few cells with spindle–associated abnormalities were found to be in the mitotic late phases of anaphase or telophase. This suggests that in viral episome-containing cells, only a minor proportion of cells with metaphase spindle deformity can bypass the metaphase – anaphase checkpoint and thus exit into anaphase and telophase. These data are consistent with findings in cells expressing only the HPV16 E6 and E7 oncoproteins, where both E6 and E7 functions contribute to the induction of mitotic spindle abnormalities, including multipolar spindles, chromosome misalignment and lagging chromosomes [31]. The defects described are associated with inactivation of the mitotic spindle assembly checkpoint that ensures engagement of all kinetochores with the spindle prior to progression through to anaphase. The role of HPV16 E6 in the modulation of this checkpoint has been studied and associated with both p53-dependent and -independent pathways [32, 33, 37]. Notably, our data indicate that slippage through the metaphase-anaphase checkpoint is enhanced in the absence of the E6 PBM since a greater proportion of cells with abnormal spindle-associated defects are in anaphase and telophase. Thus, these data offer an explanation of the p53-independent effects previously observed.

It is well established that the E6 oncoprotein can disturb mitotic progression by interfering with multiple mitotic checkpoints [38]. The multinucleation of human keratinocytes co-expressing HPV16 E6 and E7 has been attributed to E6 oncoprotein function [35, 39], in complete agreement with our findings. Failure to protect E6-expressing cells from defective cytokinesis and therefore from becoming multinucleate is linked to p53 inactivation [34, 39], but our data show that loss of the E6 PBM further perturbs mitotic division of episome–containing cells. In addition to causing an increase in the frequency of multinucleate cells, loss of the E6 PBM function also induces an increase in the degree of multinucleation of the cells, suggesting that the viability of multinucleate cells containing E6ΔPDZ genomes is greater than that of similar cells harboring WT genomes [35].

Our findings raise the interesting notion that the function of the E6 PBM of HPV18 acts to safeguard mitotic integrity in HPV-infected cells. Preserving mitotic integrity of viral genome-containing cells would be necessary to generate viable progeny, in which viral genomes can stably segregate between daughter cells and be maintained, as well as to support viral replication and promote persistence of episomes. This hypothesis would be consistent with our previous data demonstrating that HPV18 E6ΔPDZ genome-containing cells lose episomes after extended cell passage, and are unable to support efficient viral genome establishment or viral DNA amplification upon stratification [27]. Moreover, loss of episomal maintenance also occurs in keratinocytes harboring HPV16 and 31 genomes that express a mutant E6 protein unable to target PDZ proteins [28, 40]. Interestingly, it has recently been shown that maintenance replication of the mutant HPV16 E6ΔPDZ genomes could be partially restored in immortalized keratinocytes by repression of p53 protein expression [41]. The mechanism was not disclosed, but the authors hypothesized that the E6 PBM promotes viral genome maintenance by neutralization of a p53 activity that is resistant to E6-mediated degradation. These findings imply that the balance between E6 functions in infected cells is important for episomal maintenance and future studies should be directed to understanding the p53-PDZ protein axis in the HR-HPV life cycle. Moreover, a balance between E6 and E7 functions has also been shown to be important for episomal maintenance in primary keratinocytes [42]. Therefore, it cannot be ruled out that the role of the E6 PBM might also function to mediate interactions with other HPV proteins, either directly or indirectly.

Disentangling the E6 PBM function in terms of specific PDZ targets is complicated by the very large number of PDZ proteins that have been identified to interact with E6 *via* the PBM [43, 44]. Many of these PDZ substrates are degraded by E6 via the proteasome, or have altered cellular localization in HPV positive cervical cancer cells. However, our own analyses have not identified similar effects on specific E6 PDZ substrates in the context of cells harboring complete HR-HPV genomes [45], and similar observations have been made in overexpression studies (either E6 expressed alone or together with E7) in epidermal keratinocytes of transgenic mice or in primary foreskin keratinocytes in cell culture [46, 47]. Furthermore, the interaction between E6 and PDZ proteins is most likely tightly regulated during the virus life cycle by phosphorylation of the E6 motif [18, 27]. Phosphorylation of the PBM blocks the interaction with PDZ proteins and enables E6 interaction with isoforms of the 14-3-3 proteins [18, 19, 44]. The E6ΔPDZ mutant is unable to interact with PDZ or 14-3-3 proteins. However, cells harboring a HPV18 mutant genome containing an E6 mutation that abrogates phosphorylation of the E6 PBM, thus blocking interaction with 14-3-3 isoforms, but confers constitutive targeting of PDZ proteins, retain stable replication of the viral episomes and support vegetative genome amplification [27]. Thus, this strongly indicates that the underlying mechanism of the E6 mitotic function described here involves targeting of PDZ proteins. Nevertheless, it remains to be determined what specific PDZ protein(s) may function to maintain mitotic and genomic stability.

In normal epithelia, asymmetric cell division of the basal cells is required for normal differentiation; producing two daughter cells with dissimilar fates of proliferation and differentiation. This process can become uncoupled, as in the developing mouse epidermis, where differentiating suprabasal daughter cells from basal mitotic divisions retain mitotic potential in order to rapidly form the multi-layered epidermis [48]. The orientation of the mitotic spindle has an important role to play in the symmetry of cell division; the polarity complexes Par and Scribble that contain several of the E6 PBM substrates, including DLG1 and hScrib, and the 14-3-3 proteins, have important roles in directing the positioning of the mitotic spindle in *Drosophila* epithelial cells [49–51]. Moreover, some of the products of the mammalian homologues of these genes, including the PDZ protein PAR3, are also involved in directing spindle orientation in the developing embryonic mouse epidermis [48]. Notably, disturbance of mitotic spindle polarity in the mouse epidermis led to epidermal hyperproliferation and suprabasal mitoses [48, 52]. In our previous study, we showed that disruption of the E6 PBM was linked to a reduction in the expansion of replication competent cells in the upper layers of organotypic rafts [27]. In support of this, we show here that whilst in the WT cells the majority of mitotic cells reside in the suprabasal layers, the converse occurs in the mutant E6ΔPDZ rafts, where the basal layer contains the majority of mitotic cells. Moreover, our data demonstrate that loss of the E6 PBM may perturb orientation of the mitotic spindle on organotypic raft culture, and leads to spindle defects in anaphase and telophase. One explanation for this is that in infected epithelia, HPV disturbs the polarity of mitotic cells to allow rapid suprabasal expansion, and the E6 PBM somehow contributes to the deregulation of pathways that control this polarity. Interestingly, E7 associates with the spindle protein nuclear mitotic apparatus protein [53], which is important for spindle positioning in asymmetric cell divisions [48], suggesting that both E6 and E7 may cooperate to alter mitotic cell polarity and this is necessary for viral persistence and vegetative replication.

The nature of the E6 PBM interaction with PDZ domains makes it an amenable peptide-protein interaction for inhibition by small molecules with the intention to develop novel therapeutics for the treatment of HPV–associated cancers [54, 55]. Since the E6 PBM is essential for viral replication [27], inhibition of this function may be considered a route of anti-viral intervention to treat active infections. However, the data presented here offers a note of caution to disturbing the E6 PBM function in viral genome-containing cells. Firstly, loss of this function in this context is associated with viral DNA integration [28, 29], and this is a known risk factor for progression of HR-HPV infections to malignancy. In addition, we have demonstrated that viral episome-containing cells that have lost the E6 PBM function show increased genome instability in comparison to cells carrying the WT genomes. Thus, targeting this virus-host interaction therapeutically may instead accelerate the progression of HPV infections to malignancy. Also, it is worth noting that modulation of the PKA or AKT signaling pathways that lead to phosphorylation of the E6 PBM could be an independent risk factor for malignant progression by blocking E6 PDZ interactions [19, 27, 56].

Finally, this study suggests that one role of the E6 PBM in the HPV life cycle is to maintain mitotic viability of episome-containing cells, ultimately protecting the cells and permitting viral persistence. However, this E6 motif also prevents the cells from acquiring host genome instability and aneuploidy, both of which are associated with oncogenicity. Since the E6 PBM has been associated with acquisition of malignant phenotypes associated with the late stage of carcinogenesis [13, 14], this E6 function therefore has both tumor suppressor and oncogenic roles, dependent on the cellular context.

## MATERIALS AND METHODS

### Cell culture and generation of HPV18 genome-containing cells

Primary human foreskin keratinocytes (HFK) were isolated from new-born foreskin circumcision tissue samples collected from patients attending a general practitioner's practice, with informed written parental consent (Southampton and South West Hampshire research ethics committee A approval no. 06/Q1702/45). The cells were maintained in serum free media (SFM keratinocyte growth media, Life Technologies, Paisley, United Kingdom) as previously described [57]. Low passage fibroblasts (J2-3T3) were maintained in Dulbecco's modified media supplemented with 10% bovine serum (Gibco, Thermo Fisher Scientific, Cramlington, United Kingdom) and 4 mM L-glutamine.

HFK from three different donors (#1, #2, #3) were each transfected with the recircularized wild type (WT) and mutant (E6ΔPDZ) HPV18 genomes and episome-containing cell lines established, as described previously [27, 36]. Briefly, following transfection, cells were plated onto a feeder layer of γ-irradiated J2-3T3 fibroblasts (3,000 rads) in E media containing fetal calf serum (HyClone, Thermo Fischer Scientific), epidermal growth factor (BD Biosciences, Oxford, United Kingdom) and G418 antibiotic (PAA laboratories, Yeovil, UK) [36]. The cells were maintained under drug selection for 8 days and henceforth grown in E medium without G418 antibiotic. Once cell colonies emerged, they were pooled and expanded on a fibroblast feeder layer. Presence of viral episomes in the HPV18 genome transfected cells lines was confirmed by Southern analysis of total DNA extracted from the monolayer grown cells using a [α-^32^P] dCTP-labelled HPV18 genomic probe as described previously [36].

### Live cell microscopy

Real-time images of cell growth on plastic were captured by a Cell-IQ system (CM Technologies) running Imagen software version 2.8.12.0 and analyzer version 3.3.0. Cells were seeded at a density of 2 × 10^4^ cells onto a feeder layer of γ-irradiated J2-3T3 fibroblast cells in a twelve-well plate, and grown in complete E media (replaced every two days), until cell colonies were visible. The plate was then inserted into the Cell-IQ, and between three and five locations within each well were monitored every 7 min over a 16 h period at 10x magnification, in 5% CO_2_ at 37°C.

Mitotic events were scored from the resulting time-lapse videos as ‘normal’ (based upon: bipolar, visible and equal separation and timing of mitosis) or otherwise ‘abnormal’. For each cell line, between 14 and 23 colonies were counted, with every mitotic event within that colony scored, equating to 90 to 308 mitoses per cell line.

### Organotypic raft culture

Organotypic raft cultures were grown at the air-liquid interface for 13 days and fixed with 3.7% formaldehyde, as described previously [58]. Rafts were paraffin-embedded and 4 μm sections prepared (Propath UK, Ltd., Hereford, United Kingdom).

### Phase contrast and immunofluorescence microscopy

Keratinocytes were grown on γ-irradiated J2-3T3 cells seeded into wells of a six multi-well plate and viewed by phase contrast on Axiovert 100 microscope (Zeiss). For immunofluorescence microscopy, cells were grown on γ-irradiated J2-3T3 cells seeded onto glass coverslips. Once the keratinocytes had reached 60% confluence, the feeder cells were removed with EDTA in phosphate-buffered saline (PBS) and the cells were fixed in 3.6% formaldehyde (EM-Grade, TAAB laboratories, Aldermaston, Berks, United Kingdom) for 20 min and then permeabilized using 0.1% Triton X-100 for 5 min. Fixed cells were pretreated with blocking buffer (20% v/v heat-inactivated normal goat serum, 0.1% w/v bovine serum albumin in PBS) prior to addition of anti-α-tubulin antibody at 1 in 2000 dilution (clone B512, Sigma-Aldrich) or anti-CENPA antibody at 1 in 500 dilution (3-19, Abcam, Cambridge, United Kingdom). Immune complexes were recognized by an anti-mouse Alexa 488 conjugate (Molecular Probes, Life Technologies). For staining of organotypic rafts, sections (4 μm) were first de-paraffinized and subjected to low-temperature antigen retrieval as described by us previously [59]. Sections were then incubated with blocking buffer followed by staining with antibodies to α-tubulin and HPV18 E4 antibody R424 [60], and γ-tubulin at 1 in 500 dilution (mouse monoclonal antibody, Abcam), and immune complexes detected using anti-mouse Alexa 488 or 594, and anti-rabbit Alexa 594 conjugates. Nuclei were counterstained with 4′, 6′-diamidino-2-phenylindole (DAPI), and cells and raft sections mounted in ProLong® Gold Antifade reagent (Life Technologies). Stained cells and sections were viewed on a Nikon Eclipse E600 microscope fitted with epifluorescence.

### Interphase fluorescence *in situ* hybridization

Cell suspensions were harvested after 15 min treatment with hypotonic solution (0.075 M KCl) prior to methanol:acetic acid (3:1 v/v) fixation and then the cells were dropped onto clean glass slides. CEP10 and CEP12 probes (Cytocell, Cambridge, United Kingdom) were applied to the slides under a coverslip and hybridized overnight on a HYBrite (Abbott Molecular, Maidenhaed, United Kingdom). Slides were then washed in a solution containing the DNA stain DAPI (Cytocell, Cambridge, United Kingdom) and for each cell passage, at least 200 cells with single nuclei visualized.

## SUPPLEMENTARY MATERIALS FIGURES AND TABLES












