# Supplementary material for: Mitotic control of human papillomavirus genome-containing cells is regulated by the function of the PDZ-binding motif of the E6 oncoprotein
Source: Oncotarget. 2017 Jan 3;8(12):19491–506. doi: 10.18632/oncotarget.14469 (PMC5386700; doi:10.18632/oncotarget.14469)
Supplement: Supplementary file 1 [file oncotarget-08-19491-s001.pdf]

Mitotic control of human papillomavirus genome-containing cells is regulated by the function of the PDZ-binding motif of the E6 oncoprotein

SUPPLEMENTARY FIGURES

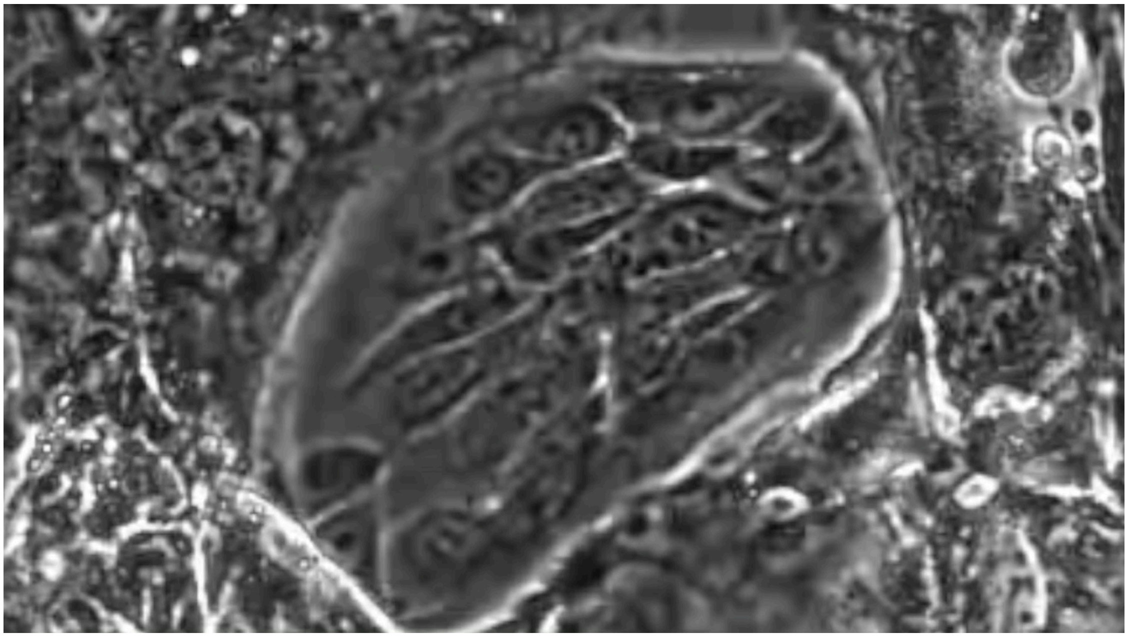

See Supplementary Video 1A

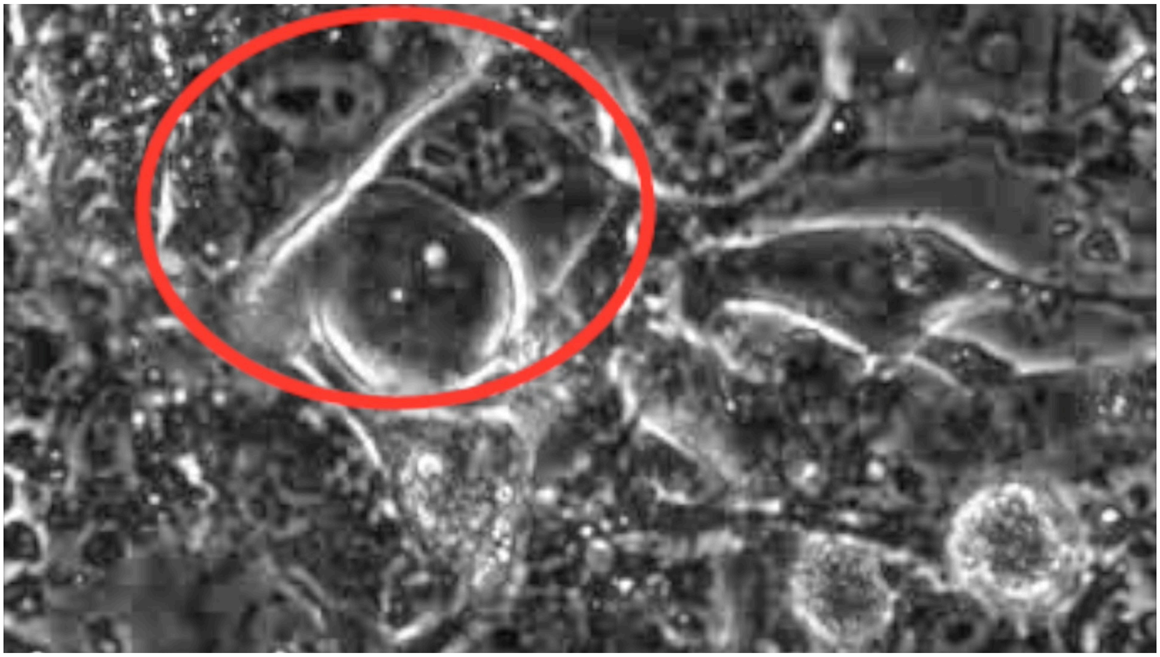

See Supplementary Video 1B

(*Continued*)

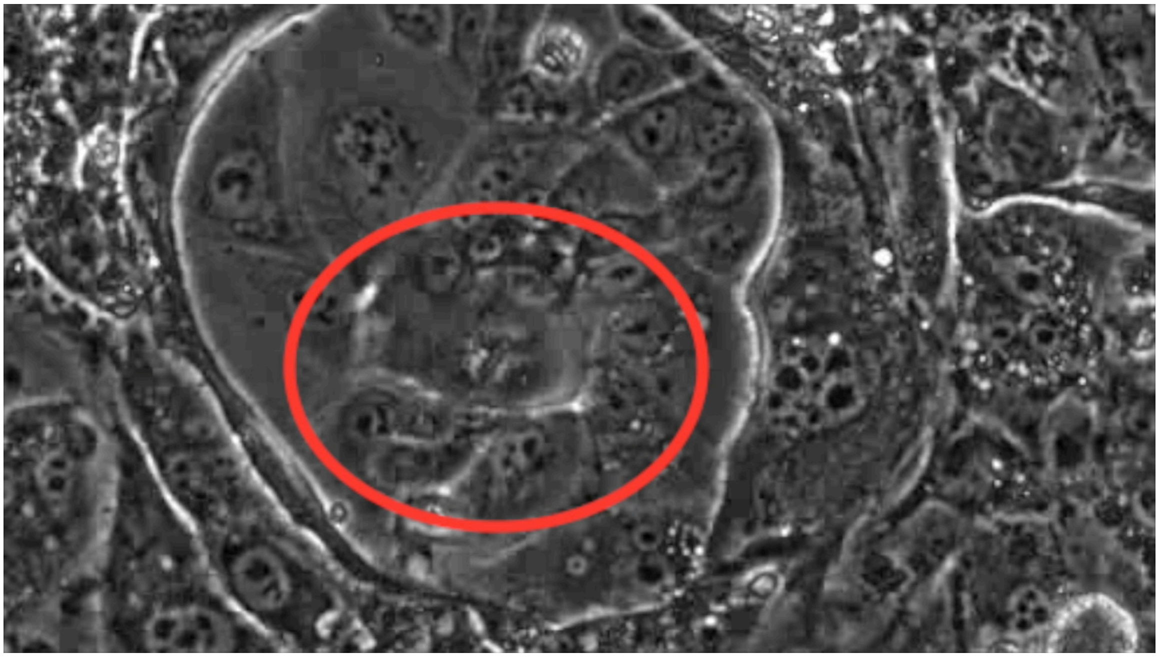

See Supplementary Video 1C

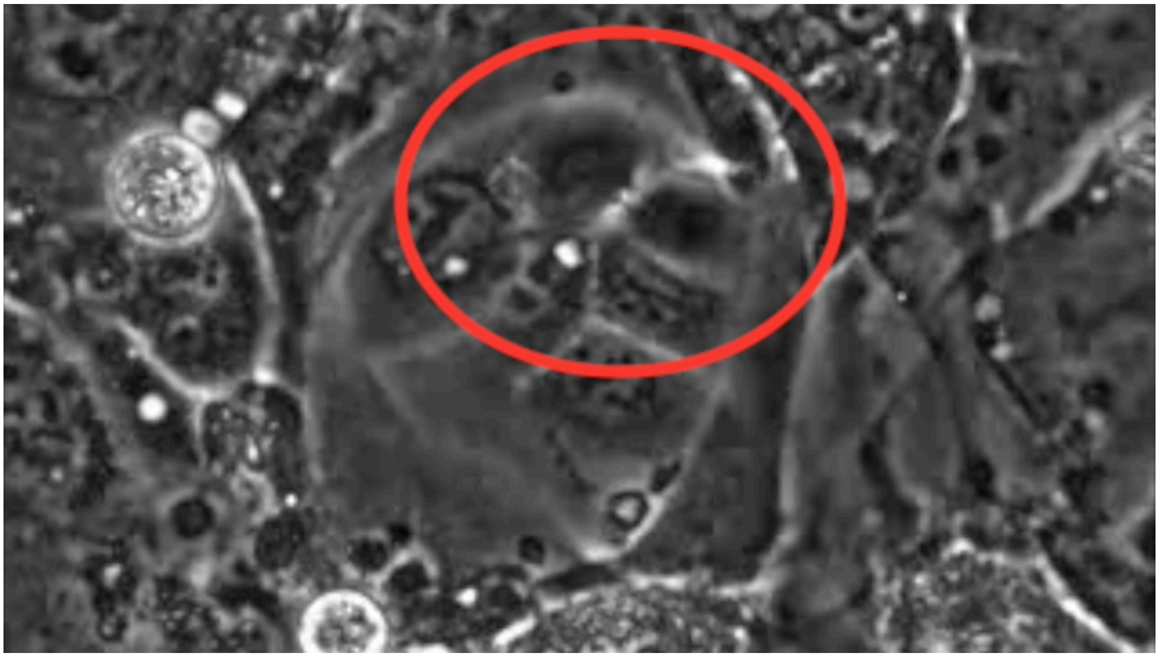

See Supplementary Video 1D

Supplementary Figure 1: Live cell microscopy of HPV18 WT and E6ΔPDZ genome-containing cells. Time-lapse movies of live cell imaging of colonies of HPV 18 genome-containing cells (WT and E6ΔPDZ) grown on γ-irradiated feeder cells. Cells within colonies that undergo normal or abnormal mitotic division and shown in the stills given in Figure 2 are identified by a red circle. S1A, E6ΔPDZ normal division; S1B, E6ΔPDZ abnormal division; S1C, WT abnormal division; S1D, WT normal division.

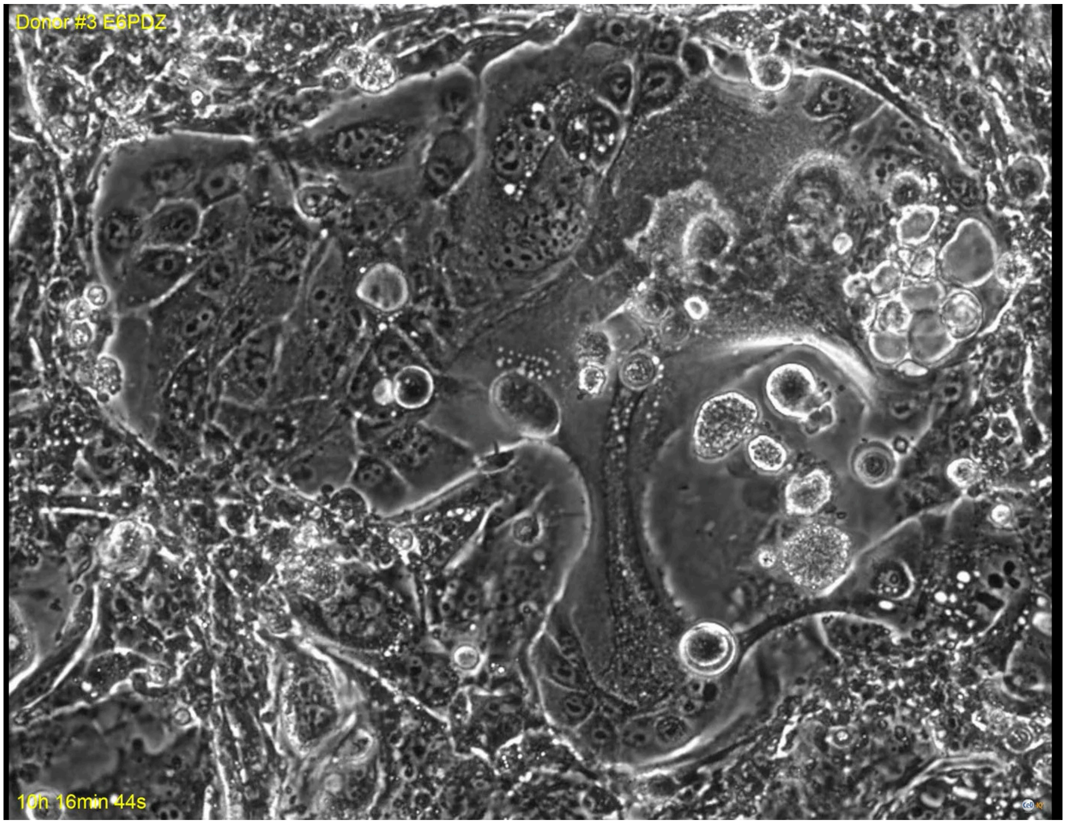

Supplementary Figure 2: Live cell microscopy of multinucleate E6ΔPDZ cells undergoing mitotic division. A time-lapse movie of live cell imaging of E6ΔPDZ cells (donor #3). The level of multinucleation is increased in E6ΔPDZ cells and these cells can enter mitosis. The example given here at the center of the colony has an elevated number of nuclei (>15) and attempts to undergo mitotic cell division.

See Supplementary Video 2
